# Clathrin expression in *Trypanosoma cruzi*

**DOI:** 10.1186/1471-2121-15-23

**Published:** 2014-06-19

**Authors:** Ligia Cristina Kalb, Yohana Camila Antunes Frederico, Cassiano Martin Batista, Iriane Eger, Stênio Perdigão Fragoso, Maurilio José Soares

**Affiliations:** 1 Laboratory of Cell Biology, Carlos Chagas Institute, Fiocruz, Rua Professor Algacyr Munhoz Mader 3775, 81350-010 Curitiba, PR, Brazil; 2 Laboratory of Molecular Biology of Trypanosomatids, Carlos Chagas Institute, Fiocruz-PR, 81350-010 Curitiba, PR, Brazil; 3 Department of General Biology, State University of Ponta Grossa, 84030-900 Ponta Grossa, PR, Brazil

**Keywords:** Clathrin, Endocytosis, Immunolocalization, *Trypanosoma cruzi*

## Abstract

**Background:**

Clathrin-mediated vesicular trafficking, the mechanism by which proteins and lipids are transported between membrane-bound organelles, accounts for a large proportion of import from the plasma membrane (endocytosis) and transport from the trans-Golgi network towards the endosomal system. Clathrin-mediated events are still poorly understood in the protozoan *Trypanosoma cruzi*, the causative agent of Chagas disease in Latin America. In this study, clathrin heavy (TcCHC) and light (TcCLC) chain gene expression and protein localization were investigated in different developmental forms of *T. cruzi* (epimastigotes, trypomastigotes and amastigotes), using both polyclonal and monoclonal antibodies raised against *T. cruzi* recombinant proteins.

**Results:**

Analysis by confocal microscopy revealed an accumulation of TcCHC and TcCLC at the cell anterior, where the flagellar pocket and Golgi complex are located. TcCLC partially colocalized with the Golgi marker TcRAB7-GFP and with ingested albumin, but did not colocalize with transferrin, a protein mostly ingested via uncoated vesicles at the cytostome/cytopharynx complex.

**Conclusion:**

Clathrin heavy and light chains are expressed in *T. cruzi*. Both proteins typically localize anterior to the kinetoplast, at the flagellar pocket and Golgi complex region. Our data also indicate that in *T. cruzi* epimastigotes clathrin-mediated endocytosis of albumin occurs at the flagellar pocket, while clathrin-independent endocytosis of transferrin occurs at the cytostome/cytopharynx complex.

## Background

Clathrin is a heteromeric protein composed of heavy chain subunits that can self-assemble into a polyhedral array on membranes. The most thoroughly characterized form of clathrin comprises light chain subunits, which are unstructured until bound to heavy chains [1]. Clathrin was described nearly 40 years ago [2] and since then the clathrin heavy chain (CHC) has been identified in all eukaryotes [3], while the clathrin light chain (CLC) subunit has proved more difficult to identify in distant eukaryotic species [1].

Clathrin-mediated vesicular trafficking is the most highly characterized mechanism by which proteins and lipids are transported between membrane-bound organelles, being responsible for a large proportion of import from the plasma membrane (endocytosis) and transport from the trans-Golgi network (TGN) towards the endosomal system [4,5]. The amino acid sequence of CHC is highly conserved from yeasts to humans, with the majority of variations representing conservative replacements or infrequent gaps, usually of less than three residues. The strong pressure to preserve the primary structure of CHC likely reflects the highly extended interleg contacts required for assembly, as well as the large number of interactions with other proteins involved in cargo sorting and the regulation of membrane coat formation [4,6].

Endocytosis is essential for eukaryotic cell survival as a basic mechanism of ingesting macromolecules and has been thoroughly characterized in mammalian and yeast cells. Internalized molecules are later degraded in the endosomal-lysosomal system and provide important precursors for several key metabolic pathways [7]. Among parasitic protozoa, endocytosis is also important for evading host immune defenses and to support the intense proliferation of specific life cycle stages [8]. A particularly good example of these parasitic protozoa are trypanosomatids, a group that includes pathogenic parasites, such as *Trypanosoma brucei*, *T. cruzi* and different *Leishmania* species, which are all associated with severe diseases in humans.

Except for a previous study demonstrating endocytosis of ferritin via coated vesicles in *Crithidia fasciculata*[9], endocytosis in trypanosomatids has been reported predominantly in *T. brucei* and also in *T. cruzi* and *Leishmania*[8]. In most trypanosomatids endocytic activity is polarized and restricted to the flagellar pocket membrane [8,10]. In *T. brucei*, endocytic activity has also been shown to vary across the different developmental stages of the parasite [11].

Endocytic mechanisms have been studied most extensively in *T. brucei*, where clathrin-mediated endocytosis is the major route for endocytosis [12]. Clathrin was found in *T. brucei* at endocytic vesicles and post-Golgi elements, suggesting that it serves a similar range of functions to those found in higher eukaryotes [13,14]. Suppression of clathrin expression in *T. brucei* using RNA interference results in rapid lethality in the bloodstream stage, the developmental form most active for endocytosis [11]. Nevertheless, the events leading up to endocytosis (regardless of whether it is mediated by clathrin) in *T. cruzi*, the causative agent of Chagas disease in Latin America, are still poorly understood. A major obstacle occurs because these two *Trypanosoma* species exhibit markedly different life cycles, and the developmental forms that can be maintained easily in the laboratory are distinct (*T. brucei* trypomastigotes x *T. cruzi* epimastigotes), making direct comparisons difficult. Furthermore, analysis of endocytic activity in *T. cruzi* is far more complex because the epimastigote form of this parasite exhibits a second endocytic portal, the cytostome/cytopharynx complex [8,15].

Morphological observations by transmission electron microscopy have previously identified coated vesicles budding off from both the flagellar pocket and a region adjacent to the TGN in *T. cruzi* epimastigotes and trypomastigotes [16,17]. Subcellular localization of clathrin has been examined in *T. cruzi* epimastigotes by immunofluorescence, however, using a heterologous antibody against bovine clathrin. Immunolabeling was observed clustered at the anterior region of cells, where the flagellar pocket and the single Golgi complex are located [16].

In this study, we have performed a thorough analysis of the CHC and CLC genes of *T. cruzi*, characterizing their expression and localization in different developmental forms of the parasite (epimastigotes, trypomastigotes and amastigotes), using both polyclonal and monoclonal antibodies produced against *T. cruzi* recombinant proteins. Our data indicate that clathrin heavy and light chains are expressed in *T. cruzi* and that they localize to the flagellar pocket and Golgi complex region.

## Results

### TcCLC and TcCHC: in silico gene identification and domain analysis

A hypothetical gene for *T. cruzi* clathrin light chain was identified in the TritrypDB database (Tc00.1047053506211.240). This gene is 648 bp long, maps between nucleotides 466,358 and 467,005 on chromosome 40 of *T. cruzi* and encodes a hypothetical protein (TcCLC) of 216 amino acids (23.3 kDa). A comparison of amino acid sequence alignments revealed that TcCLC is conserved in trypanosomatids (Table 1), with maximal identities between 37 to 45% (90% for *T. cruzi marinkellei*)*.* The percentage identity was 32–34% for more divergent organisms, such as *H. sapiens* and *S. cerevisae*, but the length of these alignments was smaller (101 and 56 amino acids, respectively, Table 1).

**Table 1:** **Amino acid sequence comparison of *Trypanosoma cruzi *** **CLC (Clathrin Light Chain and CHC (Clathrin Heavy Chain), indicating the percentage of sequence identity and similarity (in parenthesis)**

***T. cruzi***	***T. cruzi marinkellei***	***T. brucei***	***L. donovani***	***L. mexicana***	***L. major***	***H. sapiens***	***S. cerevisae***
CHC	98 (99) sl = 1704	71 (85)^a^ sl = 1706	67 (83) sl = 1705	67 (82) sl = 1705	65 (82)^a^ sl = 1705	39 (62)^a^ sl = 1694	35 (57) sl = 1680
CLC	90 (93) sl = 214	44 (61) sl = 189	40 (56) sl = 291	45 (66) sl = 145	37 (55) sl = 161	32 (55) sl = 101	34 (51) sl = 56

^a^[16].

Sequence length (sl): number of amino acids in the aligned sequences.

The *T. cruzi* clathrin heavy chain gene has already been identified in the TritrypDB database (Tc00.1047053506167.50). This gene is 5,115 bp long, maps between nucleotides 1,104,521 and 1,109,635 on chromosome 37 of *T. cruzi* and encodes a clathrin heavy chain protein (TcCHC) of 1,704 amino acids (192.891 kDa). A comparison of amino acid sequence alignments revealed that TcCHC is highly conserved in trypanosomatids (Table 1), with maximal identities between 65 and 98%*.*

Analysis of the TcCLC gene using the pFAM database revealed that the encoded protein contains a clathrin light chain domain located between amino acids 92 and 208, while the TcCHC-encoded protein contains a clathrin heavy chain linker (amino acids 333–356), clathrin-H-link domains (amino acids 358–423) and several repeats found in clathrin and in vacuolar protein sorting-associated (VPS) proteins (amino acids 544–1579, approximately 140 amino acids per repeat).

### TcCLC and TcCHC expression in Trypanosoma cruzi

**Figure 1:**
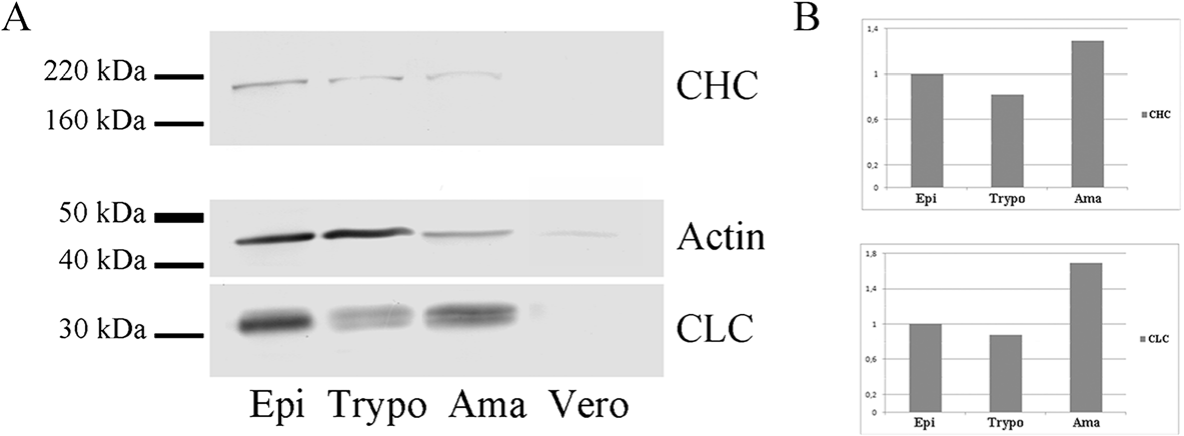
**Expression of clathrin heavy (CHC) and light (CLC) chains in different *Trypanosoma cruzi *developmental forms. (A)** Immunoblotting of parasite lysates using anti-TcCHC monoclonal IgG (upper bands), TcCLC antiserum (lower bands) and TcActin antiserum (middle bands). Epi: culture epimastigotes; Trypo: metacyclic trypomastigotes; Ama: isolated amastigotes; Vero: Vero cells (amastigote control). Each lane contained 15 μg protein. **(B)**: CHC and CLC expression, as determined by the integrated density of protein bands (normalized to TcActin), analyzed using ImageJ. To evaluate expression in amastigotes, the signal obtained for Vero cells was subtracted from that obtained for the amastigotes. Epi: culture epimastigotes in LIT medium; Trypo: metacyclic trypomastigotes; Ama: amastigotes.

A polypeptide of approximately 23.3 kDa, encoded by the entire *T. cruzi* CLC gene, was used to obtain a mouse antiserum. When assessed by western blotting, this antiserum reacted with a polypeptide of approximately 30 kDa in whole cell lysates obtained at different stages of the *T. cruzi* life cycle (Figure 1A).

A polypeptide of approximately 40 kDa, encoded by the N-terminal sequence of the *T. cruzi* CHC gene, was used to obtain polyclonal and monoclonal (IgG1/kappa isotype) antibodies. When lysates obtained at different stages of the *T. cruzi* life cycle were probed with this monoclonal antibody by western blotting, a polypeptide of approximately 190 kDa was detected corresponding to endogenous TcCHC (192 kDa). The antibody also detected the GST-TcCHC fusion protein (approximately 70 kDa) expressed in *E. coli* (data not shown). As expected, the monoclonal antibody did not detect proteins from normal *E. coli* extracts (data not shown).

Differential expression of both TcCHC and TcCLC was observed during the *T. cruzi* life cycle as determined by comparing TcCHC and TcCLC levels at different developmental stages (Figure 1B) with that of the corresponding actin loading control (Figure 1A, middle bands). Both TcCHC and TcCLC were expressed to a lesser extent in trypomastigotes and to a greater extent in amastigotes.

### Subcellular localization of TcCLC and TcCHC in Trypanosoma cruzi

**Figure 2:**
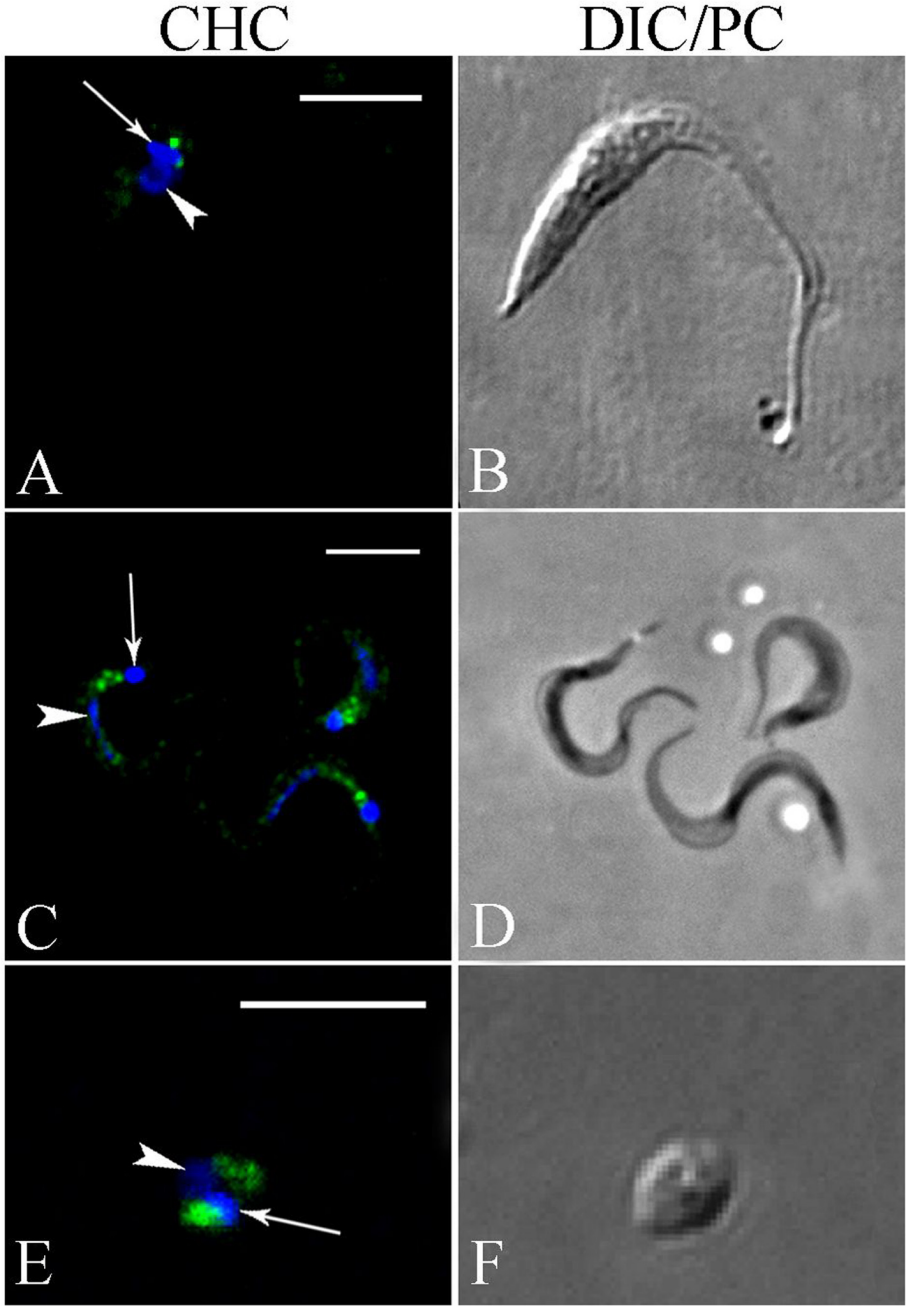
**Analysis of TcCHC localization in *Trypanosoma cruzi *using a TcCHC monoclonal antibody.** Subcellular localization of TcCHC in the various developmental forms of *T. cruzi*. **(A-B)**: epimastigote. **(C-D)**: metacyclic trypomastigotes. **(E-F)**: isolated amastigote. Note strong immunolabeling in a region adjacent to the kinetoplast (arrow), where the flagellar pocket and Golgi complex are located. Nucleus (arrowhead) and kinetoplast DNA were stained with Hoechst 33342. **A**: rabbit anti-mouse IgG conjugated to Alexa Fluor 594 (pseudocolored in green); **C** &**E**: rabbit anti-mouse IgG conjugated to Alexa Fluor 488; **B** &**F**: Differential interference contrast (DIC) images of the parasite body; **D**: phase contrast images of the parasite body. Scale bars = 5 μm.

**Figure 3:**
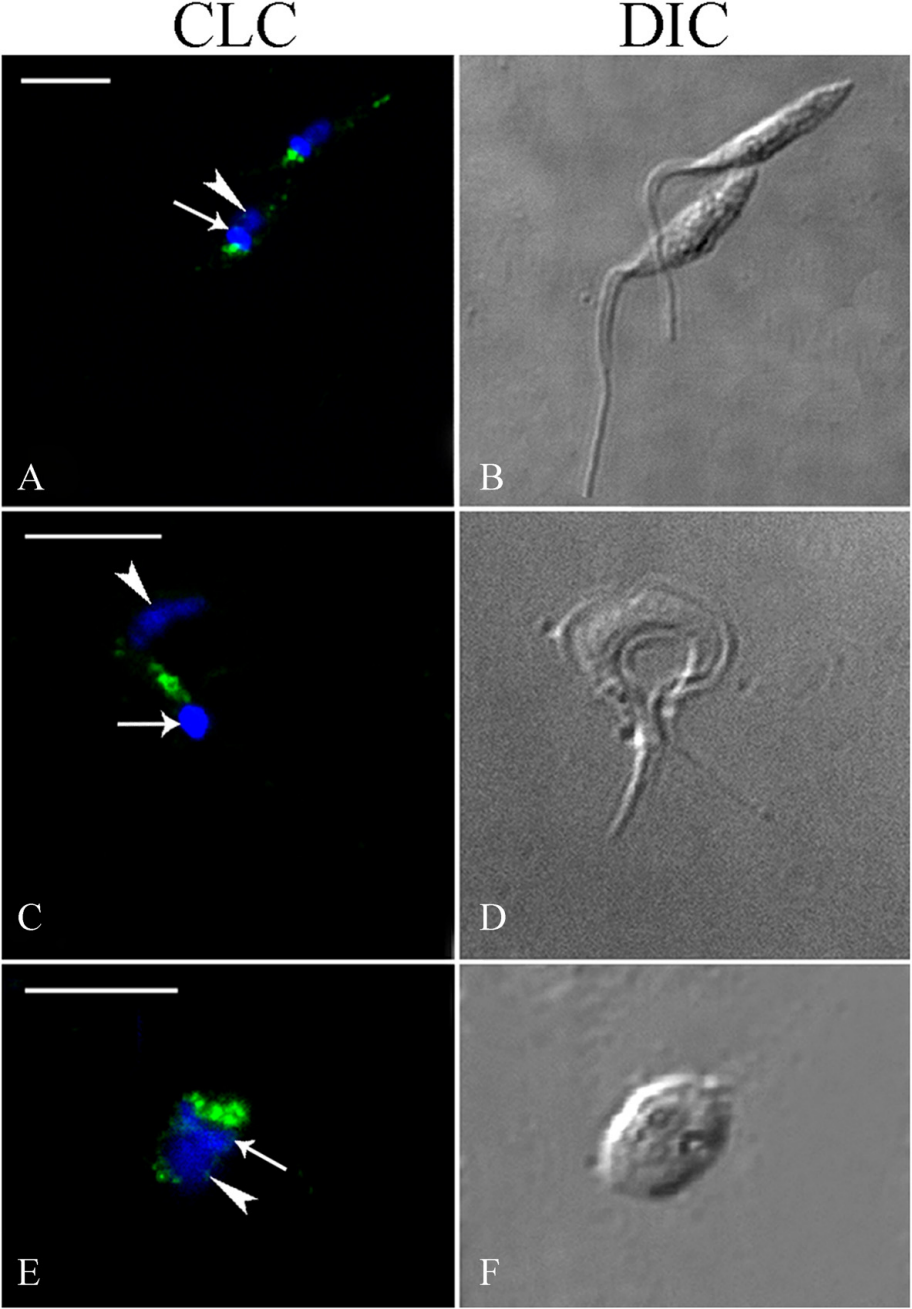
**Analysis of TcCLC localization in *Trypanosoma cruzi *using TcCLC antiserum.** Subcellular localization of TcCLC in the various developmental forms of *T. cruzi*. **(A–B)**: epimastigotes. **(C–D)**: metacyclic trypomastigote. **(E–F)**: isolated amastigote. Note the strong immunolabeling adjacent to the kinetoplast (arrow). Nucleus (arrowhead) and kinetoplast DNA were stained with Hoechst 33342. **B**, **D** &**F**: DIC images of the parasite body. Scale bars = 5 μm.

Confocal microscopy was used to visualize the cellular distribution of clathrin heavy (TcCHC) and light (TcCLC) chains in *T. cruzi*, using the TcCHC monoclonal antibody (Figure 2) and the TcCLC antiserum (Figure 3). These antibodies recognized the proteins in all developmental forms tested. Intense immunolabeling was detected adjacent to the kinetoplast, in a cell region where the flagellar pocket and Golgi complex are located.

**Figure 4:**
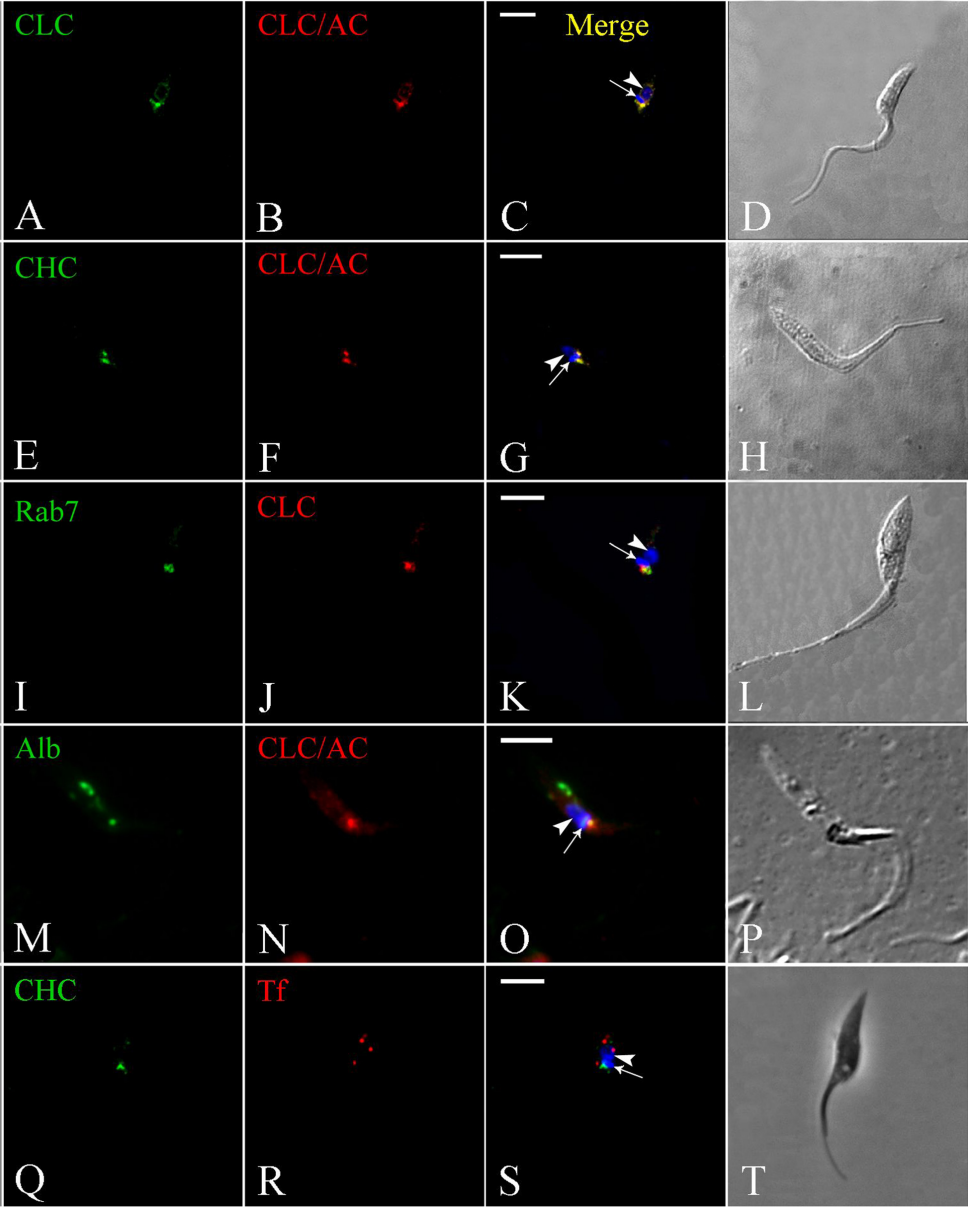
**Immunocolocalization of clathrin heavy and light chains in *Trypanosoma cruzi *epimastigotes.** Nucleus (arrowhead) and kinetoplast (arrow) DNA were stained with Hoechst 33342. **(A–D)**: Transfected epimastigote expressing CLC-A/C incubated with TcCLC antiserum **(A)** and an antibody against protein A **(B)**. **(E–H)**: Transfected epimastigote expressing CLC-A/C incubated with monoclonal antibody against TcCHC **(E)** and an antibody against protein A **(F)**. **(I–L)**: Transfected *T. cruzi* epimastigote expressing Rab7-GFP (which localizes to the Golgi complex) **(I)**, incubated with antiserum against TcCLC **(J)**; note partial colocalization of the GFP and TcCLC signals. **(M–P)**: CLC-A/C-expressing epimastigote incubated with Alexa Fluor 488-conjugated Albumin **(M)** and immunolabeled with antibody against protein A **(N)**. Note colocalization at the cell anterior, in a region corresponding to the flagellar pocket. **(Q–T)**: Wild type *T. cruzi* epimastigote immunolabeled with monoclonal antibody against TcCHC **(Q)** and incubated with Alexa Fluor 633-conjugated transferrin **(R)**; Note absence of colocalization at the cell anterior, indicating that transferrin (red) localizes to the cytostome/cytopharynx and clathrin (green) to the flagellar pocket region. **C**, **G**, **K**, **O** &**S**: Merged images. **D**, **H**, **L**, **P** &**T**: Differential interference contrast (DIC) images of the parasite body. Scale bars = 5 μm.

As mentioned above, incubation of *T. cruzi* with an antiserum against TcCLC resulted in positive immunolabeling in a region adjacent to the kinetoplast. Accordingly, this same pattern of localization was observed in TcCLC/AC-transfected *T. cruzi* epimastigotes immunolabeled with an anti-protein A antibody (Figure 4A–D; see Additional file 1). Furthermore, TcCLC colocalized with TcCHC at the anterior region of wild type epimastigotes, where the flagellar pocket and the single Golgi complex are located (Figure 4E–H).

In TcRab7-GFP transfected *T. cruzi* epimastigotes, immunolabeling with antiserum against TcCLC partially colocalized with the Golgi marker TcRab7-GFP [18] indicating that TcCLC may also localize to the flagellar pocket (Figure 4I–L). To confirm this hypothesis, wild-type epimastigotes were incubated with Alexa Fluor 488 conjugated albumin, a protein that is ingested through the flagellar pocket. As expected, TcCLC colocalized with albumin at the flagellar pocket region (Figure 4M-P; see Additional file 2), but not with albumin loaded into endocytic vesicles at the posterior cell region. Finally, TcCHC did not colocalize with Alexa Fluor 633-conjugated transferrin, a protein that is predominantly ingested via uncoated vesicles at the cytostome/cytopharynx complex and which accumulates in reservosomes (Figure 4Q–T).

The TcCLC antiserum was also used to determine the subcellular localization of endogenous TcCLC in wild-type *T. cruzi* epimastigotes by transmission electron microscopy. Ultrastructural analysis revealed immunolabeling at the flagellar pocket membrane (see Additional file 3), further confirming the results obtained by confocal microscopy.

## Discussion

Our data represent the first attempt to analyze clathrin expression and subcellular localization in the American trypanosome *T. cruzi* using homologous reagents. An *In silico* search detected a hypothetical clathrin light chain protein (TcCLC) encoded by the *T. cruzi* genome. This protein has a clathrin light chain domain, which mediates interaction with the clathrin heavy chain and regulates stabilization of clathrin coat trimerization in mammalian cells [17,19]. Amino acid sequence alignments revealed that TcCLC is conserved in trypanosomatids.

The presence of a clathrin light chain gene and expression of the encoded protein (TcCLC) have not previously been demonstrated in *T. cruzi*. In this study, we raised an antiserum against TcCLC which has an expected molecular mass of 23.3 kDa (as predicted from the cDNA sequence). However, in western blot assays the antiserum recognized a polypeptide of approximately 30 kDa in whole protein extracts of different *T. cruzi* developmental forms. A possible explanation is that the N-terminal 90 residues of TcCLC, which are rich in proline and glycine, account for the anomalous electrophoretic mobility during SDS-PAGE and consequently result in estimated molecular weights that range from 32 to 36 kDa [4].

A clathrin heavy chain gene is present in the *T. cruzi* genome [20]. Amino acid sequence alignments revealed that the encoded protein (TcCHC) is highly conserved in trypanosomatids. TcCHC has a clathrin heavy chain linker domain with an alpha-alpha superhelical structure [21]. The clathrin-H-link domain is short and often appears directly downstream from the clathrin heavy-chain linker [22]. Clathrin and VPS repeat regions are approximately 140 amino acids long, composed of multiple alpha helical repeats and occur in the arm regions of the clathrin heavy chain [23,24], as well as in VPS proteins [25].

Both clathrin light and heavy chains are present in the *T. cruzi* genome and are differentially expressed in the major developmental forms of this parasite. The low expression level observed in *T. cruzi* trypomastigotes may be related to the low endocytic activity observed for this stage [26], as opposed to the high endocytic activity found in *T. brucei* bloodstream trypomastigotes [27].

We raised monoclonal and polyclonal antibodies against the conserved N-terminal region of *T. cruzi* clathrin heavy chain. These homologous antibodies are more specific than those previously produced using clathrin isolated from bovine brain [16], which may constitute a pool of clathrin heavy and light chains. Our immunofluorescence assays demonstrated colocalization of CLC and CHC in a region adjacent to the kinetoplast of trypanosomatids, at the flagellar pocket/Golgi region. Some of these proteins localized to the Golgi complex, as demonstrated by partial colocalization with TcRab7, a Golgi marker for *T. cruzi*[18]. Accordingly, a previous study showed that *T. brucei* CLC is present at the flagellar pocket membrane, Golgi complex and endosomal compartments [28].

Evidence for clathrin-mediated endocytosis in *T. cruzi* amastigotes is provided by the positive immunolabeling for clathrin at the cell region where the small flagellum emerges. Immunolabeling was also found at the posterior region of trypomastigote forms, where both the flagellar pocket and Golgi complex are located. A previous study has shown that although transferrin can bind to the flagellar pocket membrane of trypomastigote forms, it is not endocytosed [28]. Therefore, it seems that clathrin in *T. cruzi* trypomastigotes may predominantly be involved in vesicle formation at the TGN.

TcCHC/TcCLC positive immunolabeling at the flagellar pocket region in *T. cruzi* epimastigotes indicates the occurrence of clathrin-mediated endocytosis at this site. It has previously been shown that in *T. cruzi* 80% of transferrin endocytosis occurs through the cytostome [29], while albumin endocytosis occurs at the flagellar pocket via coated vesicles [16]. Accordingly, it has been shown that transferrin uptake in *T. cruzi* is impaired following the disruption of cytostome-associated cytoskeletal elements and the stability of membrane cholesterol, but not by the disruption of clathrin-dependent endocytosis [30]. Taken together, our findings from endocytosis assays, which show colocalization of clathrin (CHC/CLC) with albumin but not with transferrin in *T. cruzi* epimastigotes, strongly suggest that in this developmental form clathrin-mediated endocytosis occurs at the flagellar pocket and that clathrin-independent endocytosis occurs at the cytostome/cytopharynx complex.

## Conclusions

Expression of clathrin heavy and light chains was demonstrated in *Trypanosoma cruzi* using homologous antibodies. Both proteins localized to the flagellar pocket and Golgi complex region of different developmental forms of this parasite (epimastigotes, trypomastigotes and amastigotes). Our data also indicate that in *T. cruzi* clathrin-mediated endocytosis occurs at the flagellar pocket, while clathrin-independent endocytosis occurs at the cytostome/cytopharynx complex. Further studies are under way to identify clathrin-associated proteins and the role these proteins play in the biology of this protozoan.

## Methods

### Reagents

Holo-transferrin, xrabbit anti-protein A antibody and bovine serum albumin were purchased from Sigma (Sigma Chemical Co, St. Louis, MO, USA). Alexa Fluor 488 and Alexa Fluor 594-conjugated rabbit anti-mouse IgGs were purchased from Molecular Probes (Carlsbad, CA, USA). AP-conjugated rabbit anti-mouse IgG was purchased from Santa Cruz Biotechnology (Santa Cruz, CA, USA). SDM79 medium, molecular weight markers (Benchmark Protein Ladder, 10–220 kDa) and the fluorescent dye Hoechst 33342 trihydrochloride were purchased from Invitrogen (Eugene, OR, USA). Alexa Fluor 488-conjugated albumin and Alexa Fluor 633-conjugated transferrin were purchased from Invitrogen (Carlsbad, CA, USA).

### Parasites

Cultured epimastigote forms of *Trypanosoma cruzi*, clone Dm28c [31], were grown at 28°C with weekly passages in liver infusion tryptose (LIT) medium [32] supplemented with 10% fetal bovine serum (FBS).

Epimastigotes were differentiated *in vitro* into metacyclic trypomastigotes under chemically defined conditions in TAU3AAG medium [33]. Briefly, epimastigotes from 5-day-old cultures (mid-log phase of growth) were harvested by centrifugation at 8,500 × g for 10 min at 10°C. The parasites were then incubated for 2 h at 28°C in triatomine artificial urine (TAU) medium (190 mM NaCl, 17 mM KCl, 2 mM MgCl_2_, 2 mM CaCl_2_, 8 mM phosphate buffer pH 6.0) at a density of 5 × 10^8^ cells/ml. This was followed by a further incubation (1:100 cell dilution) of 72 h in TAU3AAG medium (TAU supplemented with 10 mM L-proline, 50 mM L-sodium glutamate, 2 mM L-sodium aspartate, and 10 mM D-glucose). Media in culture flasks never exceeded one centimetre in depth. Metacyclic trypomastigotes were purified from 72-h-old cultures by DEAE-51 cellulose chromatography [33].

To obtain isolated amastigotes, metacyclic trypomastigotes were used to infect Vero cells (ATCC nr. CRL-1586), which were cultivated in Dulbecco’s Modified Eagle’s medium (DMEM, Sigma) supplemented with 5% FBS and incubated in a humidified atmosphere with 5% CO_2_ at 37°C. After 10 days of infection, amastigotes released into the supernatant were harvested by centrifugation at 1,000 × g for 5 min.

### In silico analysis

The TritrypDB database was searched for *T. cruzi* genes encoding amino acid sequences for clathrin heavy chain (TcCHC) and hypothetical clathrin light chain (TcCLC) proteins. Gene IDs Tc00.1047053506167.50 and Tc00.1047053506211.240 were found for TcCHC and TcCLC respectively. The corresponding amino acid sequences of TcCHC and TcCLC were aligned with the Protein Blast algorithm (Blastp-NCBI, Bethesda, MD, USA). The pFAM software (Sanger Institute, Cambridge, UK) was used for domain analysis.

### Cloning and expression of *Trypanosoma cruzi* TcCHC and TcCLC

A fragment corresponding to the first 1,360 nucleotides of the TcCHC open reading frame (ORF) was amplified by PCR using the forward prime: 5′-ATGAACGGCCCCTTGACGACA-3′ and reverse primer: 5′-TGACAGACCCACCTTTCGGCACAAC-3′. The amplified product was cloned into the *Sal*I and *Not*I sites of the pGEX-4 T1 vector (GE Healthcare Bio-Sciences, Pittsburgh, PA, USA), resulting in the incorporation of a GST (Glutathione-S-Transferase) tag upstream of the insert.

The complete coding sequence of TcCLC was amplified using synthetic primers based on the *T. cruzi* CL Brener sequence, available from the *T. cruzi* genome database ( http://www.genedb.org). The putative 648 bp ORF, encoding a protein of 23.3 kDa, was amplified by PCR using genomic DNA as template and forward and reverse primers (forward primer: 5′-**GGGGGATCC**ATGGACCCTTTTGAAGGAAGC-3′, reverse primer: 5′-**GGGGTCGAC**TTATTGAGCGGTTTCGCCCT-3′) which incorporated flanking *Bam*HI and *Sal*I restriction sites (in bold) into the amplified product for subsequent insertion into the pGEX-4T1vector.

Positive TcCHC and TcCLC clones were purified with a QIAprep Spin Miniprep kit (Qiagen, Valencia, CA, USA) and sequencing was performed at Macrogen Inc. (Seoul, Korea). The correct sequence was confirmed with the BLAST algorithm. The amplified fragments were inserted into the pGEX-4 T1 vector and used to transform the *Escherichia coli* TOP10 strain (Invitrogen, USA). Production of recombinant proteins was induced following addition of 0.3 mM IPTG (isopropyl-β-D-1-thiogalactopyranoside) to bacterial cultures. Inclusion bodies containing the TcCHC recombinant protein were isolated from the insoluble fraction and the recombinant protein then purified by electro-elution. TcCLC recombinant protein was purified from the soluble fraction using a GST column.

To generate transfected epimastigotes expressing TcCLC with a protein A and C amino-terminal fusion (TcCLC/AC), TcCLC cDNA was cloned into the pTcGWPTP expression vector. The pTcGWPTP vector encodes proteins A and C and is a modification of the previously described pTcGWGFP vector [34]. The TcCLC gene was used to design forward (5′-ATGGACCCTTTTGAAGGAAGC-3′) and reverse (5′-TTATTGAGCGGTTTCGCCCT-3′) primers flanked by sequences compatible with the Gateway (Invitrogen, USA) cloning platform to enable subsequent subcloning into the target vector. The resulting pTcGWPTP plasmid encoding the TcCLC gene fused to proteins A and C was used to transfect parasites.

### Parasite transfection

*T. cruzi* epimastigote cultures were grown at 28°C in LIT medium supplemented with 10% FBS to a density of approximately 3 × 10^7^ cells/ml. Parasites were then harvested by centrifugation at 3,000 × g for 5 min at room temperature, washed once in phosphate-buffered saline (PBS, pH 7.2) and resuspended in 0.4 ml of electroporation buffer (140 mM NaCl, 25 mM HEPES, 0.74 mM Na_2_HPO_4_, pH 7.5) at a density of 1 × 10^8^ cells/ml. Cells were then transferred to a cuvette (0.2 cm gap width) and 10–15 μg DNA added. The mixture was placed on ice for 10 min and then subjected to two pulses of 450 V/500 μF using the Gene Pulser II (Bio-Rad, Hercules, CA, USA). Following electroporation, cells were cultured in 10 ml LIT medium containing 10% FBS and incubated for 24 h at 28°C. The antibiotic G418 (500 μg/ml) was then added to the culture medium and stable, resistant cells were obtained approximately 20 days after transfection. Stably transfected cells were maintained in cultures containing 250 μg/ml G418.

### Antibody (Ab) production

Polyclonal antibodies raised against recombinant TcCHC and TcCLC proteins were produced in albino Balb/c mice. Approximately 50 μg of purified GST-tagged protein was mixed at a ratio of 1:1 with complete Freund’s adjuvant and inoculated intra-peritoneally. After 15 days the animals received three consecutive boosts with an additional 20 μg of the antigen and 77 μl of Alu-Gel adjuvant (Serva, Heidelberg, Germany), at 2-week intervals. Antiserum was obtained 5 days after the last booster injection. A monoclonal antibody against TcCHC was produced from spleen cells isolated from a responsive mouse, as described previously [35].

All animals were handled in strict accordance with the recommendations made in the Guide for Animal Use of the Fundação Oswaldo Cruz (Fiocruz, Brazil). The protocol was approved by the Committee on Animal Experimentation (CEUA/FIOCRUZ), number P-47/12-3.

### Western blot analysis

For immunoblotting, total protein extracts of *T. cruzi* epimastigotes, metacyclic trypomastigotes and isolated amastigotes, as well as whole extracts of Vero cells, were obtained by cell lysis by resuspending PBS-washed cells (1 × 10^6^ cells/μl) in sodium dodecyl sulfate-polyacrylamide gel electrophoresis (SDS-PAGE) sample buffer. Proteins (15 μg/lane) were fractionated by SDS-PAGE in gradient polyacrylamide gels and transferred onto nitrocellulose membranes (Hybond C, Amersham Biosciences, Buckinghamshire, United Kingdom) according to standard protocols [36]. Following protein transfer, the membranes were blocked with 5% non-fat milk/0.05% Tween-20 in PBS and incubated for 1 h with blocking buffer containing either the antiserum raised against the TcCLC protein (1:200 dilution) or the monoclonal antibody against TcCHC. A polyclonal anti-TcActin antibody [37] was used as a loading control. After 3 washes with 0.05% Tween-20/PBS, the membranes were incubated for 1 h with alkaline phosphatase-conjugated rabbit anti-mouse IgG (1:10,000 dilution), washed three times with 0.05% Tween-20/PBS and bands then visualized using BCIP-NBT (Promega, USA) solution.

### Immunofluorescence assays

Parasites were washed and resuspended at 1 × 10^7^ cells/ml in PBS. The cells were fixed for 30 min with 4% paraformaldehyde at room temperature, washed twice in PBS and then adhered to 0.1% poly-L-lysine-coated coverslips following 20 min incubation at room temperature. Cells were permeabilized with 0.5% Triton X-100 in PBS for 5 min, washed with PBS, blocked for 1 h with 1.5% bovine serum albumin in PBS and then incubated for 1 h with antiserum (1:150) for TcCLC or TcCHC, or with monoclonal Ab (undiluted) for TcCHC. After washing, samples were incubated with Alexa Fluor 488 or 594-conjugated rabbit anti-mouse IgG at a 1:600 dilution. Nuclear and kinetoplast DNA were stained with Hoechst 33342. After extensive washes, the coverslips were mounted onto glass microscope slides with mounting medium containing N-propyl-gallate as an anti-fade agent. The samples were examined using a Leica SP5 confocal laser-scanning microscope (Leica Microsystems, Mannheim, Germany) at the Microscopy Facility of the Institute Carlos Chagas, Fiocruz-PR. Acquired images were processed to improve contrast using Adobe Photoshop CS5 (Adobe Systems Incorporated, USA).

For colocalization of endogenous TcCLC and TcCHC with exogenously expressed TcCLC/AC in transfected *T. cruzi* epimastigotes, 3-day-old cells were washed twice with PBS, fixed for 30 min with 4% paraformaldehyde and incubated for 1 h at 37°C with either TcCLC antiserum (1:150), protein A antibody (1:40,000) or TcCHC monoclonal antibody. After three washes in PBS, the samples were incubated under the same conditions with a secondary Alexa Fluor 488-conjugated rabbit anti-mouse antibody (1:600) or an Alexa Fluor 594-conjugated goat anti-rabbit antibody (1:600). A negative control was performed by incubating protein A antibody with wild-type epimastigotes (data not shown). Samples were further processed and imaged as described above.

To assess colocalization of endogenous TcCLC with the Golgi complex we used TcRab7-GFP transfected *T. cruzi* epimastigotes. It has been shown that TcRab7 is a Golgi marker for *T. cruzi*. A plasmid vector encoding TcRab7 fused to GFP (kindly provided by Michel Batista, Instituto Carlos Chagas/FIOCRUZ-Paraná) was used to obtain TcRab7-GFP transfected *T. cruzi* epimastigotes, as previously described [34]. TcRab7-GFP transfected cells were fixed and incubated with antiserum against TcCLC as described above.

### Endocytosis assay

*T. cruzi* epimastigotes were washed twice with PBS and then subjected to a nutritional stress in PBS for 15 min at 16°C. Epimastigotes were then incubated for 30 min at 16°C with Alexa Fluor 633-conjugated transferrin or Alexa Fluor 488-conjugated albumin (1:20 dilution for both) to allow uptake of the labelled proteins. To assess the colocalization of transferrin with TcCHC or albumin with TcCLC, parasites were fixed for 30 min with 4% paraformaldehyde, permeabilized for 5 min with 0.5% Triton in PBS and incubated with either TcCHC monoclonal antibody or protein A antibody (1:40,000). The samples were washed three times with PBS and then incubated with rabbit anti-mouse secondary antibody coupled to Alexa Fluor 488 (1:600) or Alexa Fluor 594. The samples were washed three times with PBS, incubated for 5 min with Hoechst 33342 and examined using a Leica SP5 confocal laser-scanning microscope.

### Transmission electron microscopy

Five-day-old *T. cruzi* epimastigote cultures were collected by centrifugation and fixed for 1 h with 0.1% glutaraldehyde/4% paraformaldehyde in 0.1 M phosphate buffer (pH 7.2). The cells were then washed in 0.1 M cacodylate buffer (pH 7.2), dehydrated in a series of graded ethanol washes and infiltrated overnight with a 1:1 dilution of ethanol 100%: Lowicryl K4M Monostep resin (EMS, Washington, PA, USA). After embedding for 6 h in pure resin, the samples were polymerized for 48 h at -20°C under UV light. Ultra-thin sections were collected on nickel grids, incubated for 30 min with 50 mM ammonium chloride in PBS (pH 7.2) and then incubated for 1 h with TcCLC antiserum diluted at 1:50 in PBS/1.5% albumin/0.01% Tween 20 (washing buffer). After washing, the grids were incubated for 1 h with a rabbit anti-mouse antibody conjugated to 10-nm gold particles (diluted 1:50 in washing buffer). Additional washes in buffer and distilled water were performed before the grids were stained for 30 min with 5% uranyl acetate and for 2 min with lead citrate. Samples were observed in a JEOL 1200EXII transmission electron microscope at the Electron Microscopy Center, Federal University of Paraná, Curitiba-PR.

## Competing interests

The authors declare that they have no competing interests.

## Authors’ contributions

LCK performed the TcCHC experiments, acquired and analyzed the fluorescence and transmission electron microscopy data and drafted the manuscript. YCAF performed the TcCLC experiments. CMB carried out the bioinformatic analysis and helped with the endocytosis assays. IE helped with monoclonal antibody production. SPF participated in study design and helped with cloning and expression of *Trypanosoma cruzi* TcCHC and TcCLC. MJS conceived the study, participated in its design and coordination and edited the final version of the manuscript. All authors approved the final manuscript.

## Supplementary Material

Additional file 1 **Immunofluorescence assay to demonstrate that monoclonal antibodies do not bind protein A in transfected *T. cruzi *** **epimastigotes expressing TcCLC fused with protein A/C. A)** As a control, *T. cruzi* transfected epimastigotes were first incubated with Protein A antibody. Positive immunolabeling was observed at the cell anterior, in close proximity to the kinetoplast. **B)** Transfected epimastigotes incubated with a monoclonal antibody against TcCruzipain [38], which specifically recognizes the *T. cruzi* reservosomes at the cell posterior (arrow). **C)** Transfected epimastigotes incubated with a monoclonal antibody against TcNup [39], which specifically recognizes a protein associated to the nuclear membrane of *T. cruzi*. As expected, monoclonal antibodies in B and C were specific for their target proteins, but did not recognize CLC-A/C at the cell anterior. These results show that monoclonal antibodies do not cross react with the protein A/C tag in our transfected epimastigotes. Scale bar = 5 μm. Click here for file

Additional file 2 ***T. cruzi *** **epimastigotes incubated for 30 min at 16°C with Alexa Fluor 488-conjugated albumin and subsequently with anti TcCruzipain monoclonal antibody (detected with Alexa Fluor 594-conjugated secondary antibody).** Albumin accumulates at the cell anterior, perpendicular to the kinetoplast (k), in a location that corresponds to the flagellar pocket region. Furthermore, albumin also localizes to the cell posterior, behind the nucleus (n). However, at 16°C albumin does not colocalize with TcCruzipain (a reservosomal marker). Therefore, this posterior labeling represents endocytic vesicles en route to reservosomes. Scale bar = 5 μm. Click here for file

Additional file 3 **Ultrastructural immunolocalization of clathrin light chain in *T. cruzi *** **epimastigote by transmission electron microscopy.** Gold labeling (arrow) is found associated with the flagellar pocket membrane. F: flagellum; K: kinetoplast. Scale bar = 100 nm. Click here for file
