# Supplementary figures and images for: Clathrin expression in Trypanosoma cruzi
Source: BMC Cell Biol. 2014 Jun 19;15:23. doi: 10.1186/1471-2121-15-23 (PMC4073184; doi:10.1186/1471-2121-15-23)

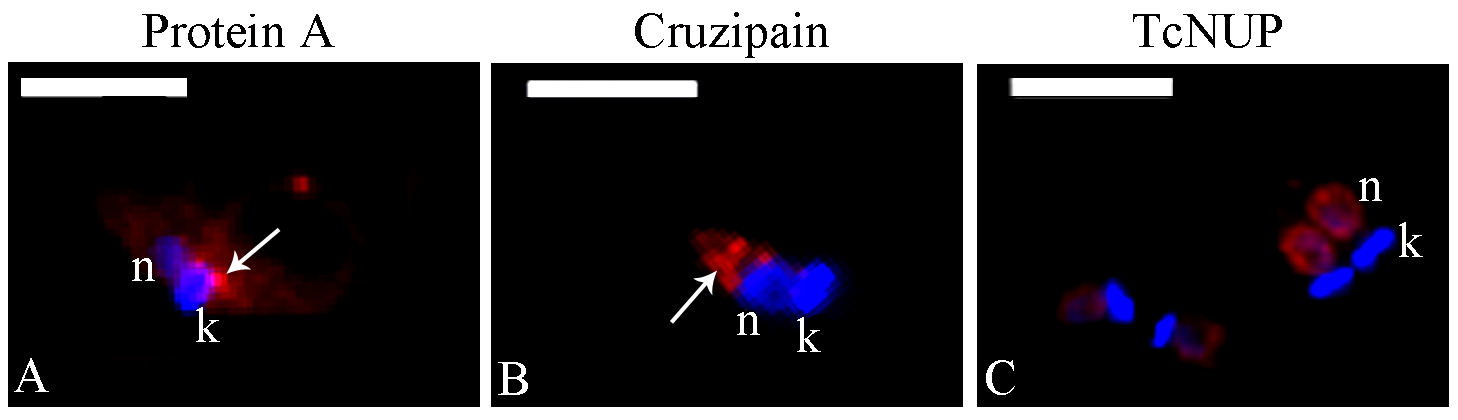

Supplement: Additional file 1 — Immunofluorescence assay to demonstrate that monoclonal antibodies do not bind protein A in transfected T. cruzi epimastigotes expressing TcCLC fused with protein A/C. A) As a control, T. cruzi transfected epimastigotes were first incubated with Protein A antibody. Positive immunolabeling was observed at the cell anterior, in close proximity to the kinetoplast. B) Transfected epimastigotes incubated with a monoclonal antibody against TcCruzipain [38], which specifically recognizes the T. cruzi reservosomes at the cell posterior (arrow). C) Transfected epimastigotes incubated with a monoclonal antibody against TcNup [39], which specifically recognizes a protein associated to the nuclear membrane of T. cruzi. As expected, monoclonal antibodies in B and C were specific for their target proteins, but did not recognize CLC-A/C at the cell anterior. These results show that monoclonal antibodies do not cross react with the protein A/C tag in our transfected epimastigotes. Scale bar = 5 μm. [file 1471-2121-15-23-S1.tiff]

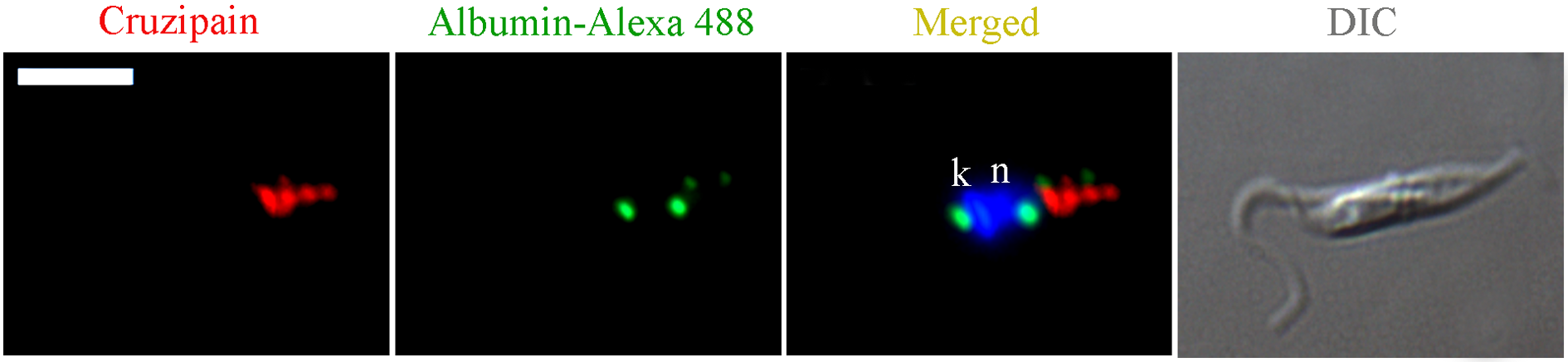

Supplement: Additional file 2 — T. cruzi epimastigotes incubated for 30 min at 16°C with Alexa Fluor 488-conjugated albumin and subsequently with anti TcCruzipain monoclonal antibody (detected with Alexa Fluor 594-conjugated secondary antibody). Albumin accumulates at the cell anterior, perpendicular to the kinetoplast (k), in a location that corresponds to the flagellar pocket region. Furthermore, albumin also localizes to the cell posterior, behind the nucleus (n). However, at 16°C albumin does not colocalize with TcCruzipain (a reservosomal marker). Therefore, this posterior labeling represents endocytic vesicles en route to reservosomes. Scale bar = 5 μm. [file 1471-2121-15-23-S2.tiff]

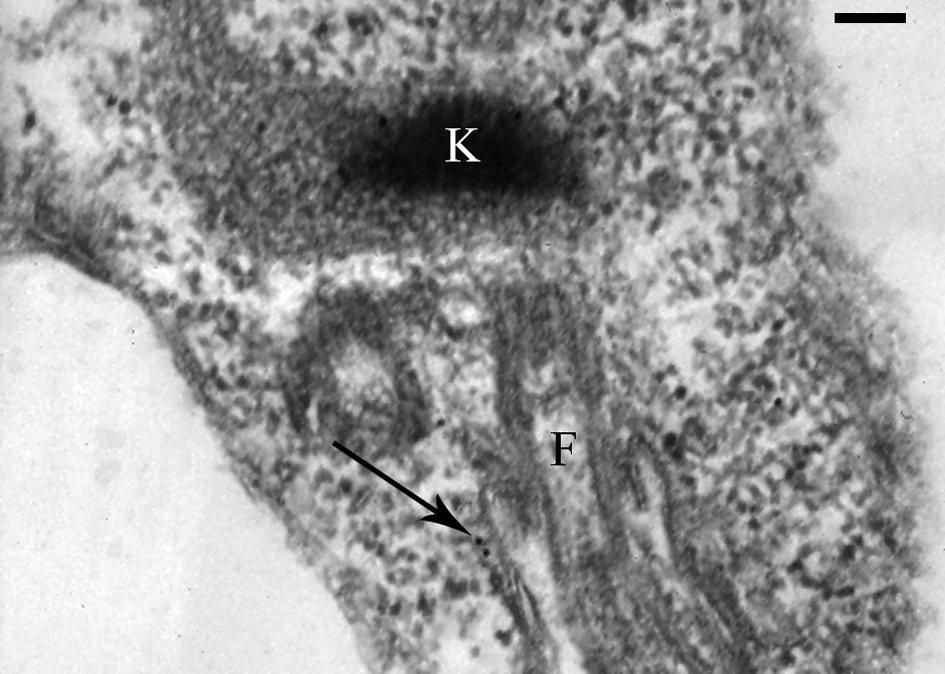

Supplement: Additional file 3 — Ultrastructural immunolocalization of clathrin light chain in T. cruzi epimastigote by transmission electron microscopy. Gold labeling (arrow) is found associated with the flagellar pocket membrane. F: flagellum; K: kinetoplast. Scale bar = 100 nm. [file 1471-2121-15-23-S3.tiff]
